# Performance of HPV genotyping and *GYPC* methylation as a triage strategy for HPV-positive women with normal or minimal cytological findings

**DOI:** 10.3389/fmed.2025.1575887

**Published:** 2025-04-17

**Authors:** Saiping Mao, Xing Fan, Li Yang, Hongtao Li, Dandan Liu, Xiaoli He, Xiaoli Wang, Fang Yu

**Affiliations:** ^1^Department of Obstetrics and Gynecology, Changsha Hospital for Maternal and Child Health Care Affiliated to Hunan Normal University, Changsha, China; ^2^Hunan Normal University, Changsha, China; ^3^Department of Medicine, Hunan Hoomya Gene Technology Co., Ltd., Changsha, China

**Keywords:** cervical screening, cervical intraepithelial neoplasia, *GYPC* methylation, high-risk human papillomavirus, absolute risk

## Abstract

**Objective:**

High-risk human papillomavirus (HR-HPV) screening has increased colposcopy referrals, particularly for women with HR-HPV positivity but no intraepithelial lesion or malignancy (NILM) and those with atypical squamous cells of undetermined significance (ASC-US). A fraction of low-grade squamous intraepithelial lesions (LSILs) is associated with cervical intraepithelial neoplasia grade 2 or worse (CIN2+) during diagnosis. This study evaluated the ability of *GYPC* methylation (*GYPC^m^*) to distinguish between <CIN2 and CIN2 + in HPV-positive women with NILM, ASC-US, and LSIL cytology. It also assessed the absolute CIN2+/CIN3 + risk of the triage strategies *GYPC^m^*, HPV genotyping, and their combination and compared the clinical performance of each triage strategy.

**Methods:**

To improve cervical screening efficiency, risk stratification based on HPV genotyping and *GYPC^m^* was used as a triage strategy.

**Results:**

*GYPC^m^* distinguished between <CIN2 and CIN2 + with an area under the receiver operating characteristic curve (AUC) of 0.828. The CIN2 + risk for *GYPC^m^* (+) was 36.2%, while that for *GYPC^m^* (−) was 2.3%. HPV16/18 combined *GYPC^m^*, (+) and (+), (−) and (+) with absolute CIN2 + risk was 41.2 and 35.1%, respectively, whereas (+) and (−), (−) and (−), absolute CIN2 + risk was 6.0 and 1.5%, respectively. Colposcopy referral rates for HPV16/18 or *GYPC^m^* and HPV16/18 or ASC-US+ were 35.6 and 79.2%, respectively, with concordant sensitivities (90.2% vs. 87.8%, *p* > 0.999) and significant differences in specificity (70.5% vs. 21.8%, *p* < 0.001). The HPV16/18 or *GYPC^m^* triage strategy required the least number of referrals to detect a CIN2 + at 3.9 (3.3–4.6).

**Conclusion:**

HPV16/18 or *GYPC^m^* as a triage tool in HPV-positive women with NILM, ASC-US, and LSIL cytology significantly reduced colposcopy referrals while maintaining sensitivity similar to that of HPV16/18 or ASC-US+.

## 1 Introduction

Human papillomavirus (HPV)-based testing is more sensitive but less specific than cytology-based testing for detecting high-grade cervical intraepithelial neoplasia (CIN) and cervical cancer (1, 2). Effective triage of HPV-positive women is crucial to prevent unnecessary colposcopy referrals (3). However, triaging HPV-positive women with cytology and/or HPV16/18 genotyping leads to an approximately 4-fold increase in colposcopy referrals across both screening rounds compared to relying solely on cytology-based screening (4).

The latest Chinese guidelines recommend either HR-HPV (high-risk HPV) primary screening or a combination of cytology and direct colposcopy for HR-HPV-positive women with atypical squamous cells of undetermined significance (ASC-US) or low-grade squamous intraepithelial lesion (LSIL), or more severe conditions (ASC-US+) (5, 6). For HR-HPV-positive cases with cytology indicating no intraepithelial lesion or malignancy (NILM) and presenting clinical symptoms, a referral for colposcopy in outpatient clinics is also recommended (5). However, the cytology screening program has been superseded by a primary HR-HPV-based screening, accompanied by cytology triage (7). The screening strategy has led to higher clinically relevant costs, primarily caused by increased colposcopy referrals and detection of ≤CIN1 (8, 9). The primary reason for the increase in colposcopy referrals is the presence of a few high-grade cervical intraepithelial neoplasia (CIN2+) in HR-HPV-positive patients with NILM, ASC-US, and LSIL cytology results (9, 10). Management of risk stratification using highly specific molecular markers for women with HR-HPV and minor or no cytology abnormalities may reduce the number of colposcopy referrals while maintaining clinical sensitivity.

Cervical cancer is caused by persistent HPV infection (11). Different HPV genotypes carry varying CIN3 + risks, with HPV16/18 genotyping considered the most effective cervical cancer-reducing triage option for HR-HPV-positive women (12). Another strategy that has been explored in many recent studies involves the analysis of DNA methylation in host cell genes. Hypermethylation of the promoter region of a tumor suppressor gene is a critical step in cervical carcinogenesis (13). Methylation levels correlate positively with the duration of HR-HPV infection and the severity of CIN, ultimately reaching significantly high levels in cervical cancer (14, 15). Overexpression of methylation and underexpression of mRNA in *Glycophorin C* (*GYPC*) have been observed in several cancers, carrying significant diagnostic and prognostic implications (16–18). In HR-HPV-positive women, the odds ratios for CIN2 + in *GYPC* with high and moderate methylation, relative to hypomethylation, were 23 and 61, respectively, making it a highly specific and objective molecular indicator (19).

In this study, we evaluated the performance of *GYPC* methylation (*GYPC^m^*), HPV16/18 genotyping, and their combination in HR-HPV-positive women with NILM, ASC-US, and LSIL cytology.

## 2 Methods

### 2.1 Participants

**Figure 1:**
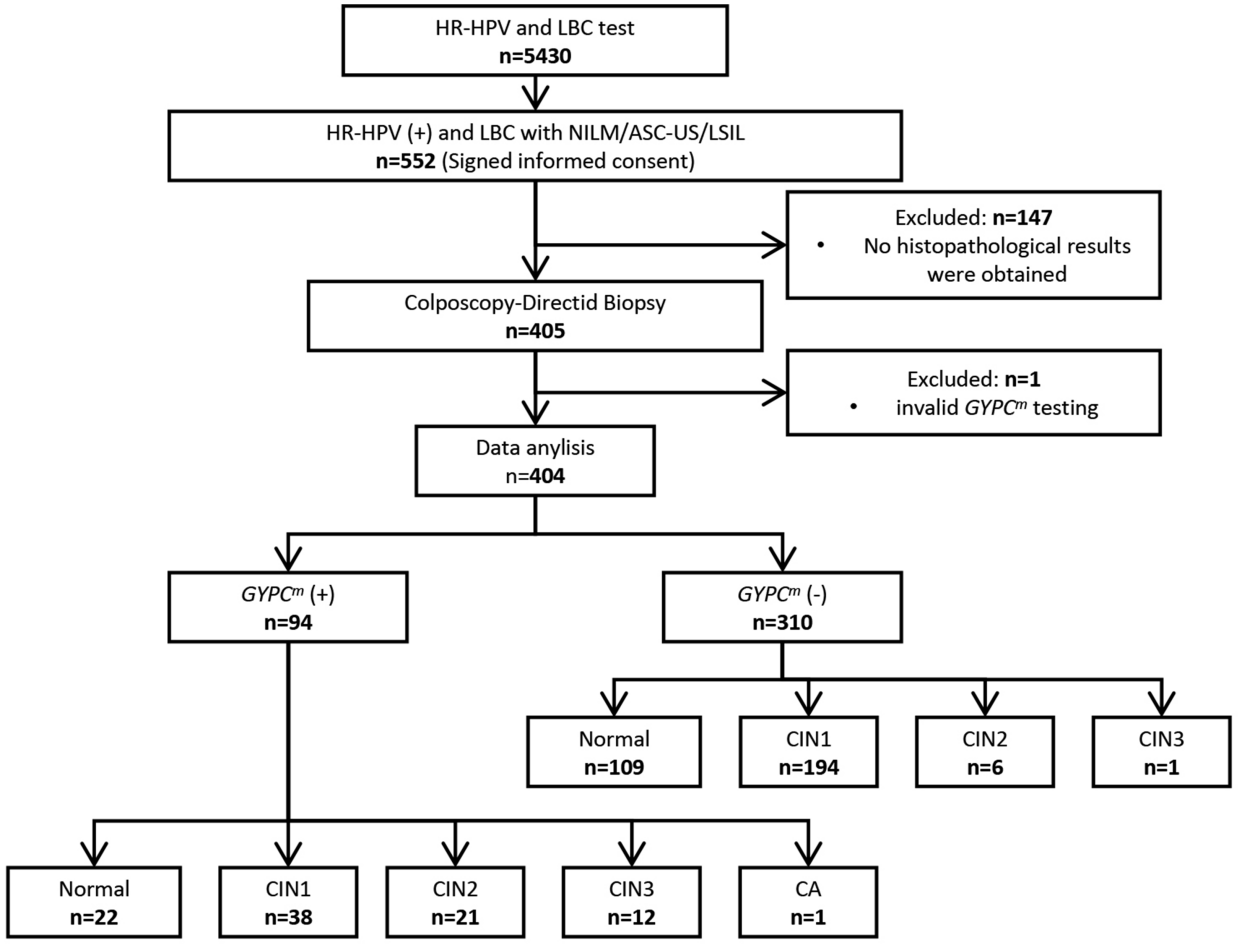
Study flowchart. HR-HPV, high-risk human papillomavirus; LBC, liquid-based cytology; NILM, no intraepithelial lesion or malignancy; ASC-US, atypical squamous cells of undetermined significance; LSIL, low-grade squamous intraepithelial lesion; *GYPC^m^*, *GYPC* methylation; CIN1, cervical intraepithelial neoplasia grade 1; CIN2, cervical intraepithelial neoplasia grade 2; CIN3, cervical intraepithelial neoplasia grade 3; CA, cervical adenocarcinoma.

This cross-sectional observational study recruited HR-HPV-positive women with NILM, ASC-US, and LSIL cytology and used the cervical exfoliated cells for *GYPC^m^* testing to assess triage performance. Among those recruited, NILM cytology combined with non-HPV16 infection was clinically indicated for colposcopy referral. The inclusion criteria were as follows: (1) women older than 30 years, (2) HR-HPV-positive, and (3) women with NILM, ASC-US, or LSIL within 3 months of HPV testing. The exclusion criteria were as follows: (1) pregnant women, (2) patients who had undergone surgery for cervical lesions, (3) patients who had received treatment for cervical and other cancers, and (4) immunocompromised individuals. Figure 1 shows the flowchart of the study design. A total of 5,430 women underwent HR-HPV testing and liquid-based cytology (LBC) between June 2023 and June 2024 at gynecologic outpatient clinics. Among these, 552 women were HR-HPV-positive with cytologic results of NILM, ASC-US, or LSIL. Of these, 147 did not undergo colposcopic biopsy, 1 had an invalid *GYPC^m^* test, and 404 were included in the statistical analysis. This study was approved by the Research and Clinical Trial Ethics Committee of Changsha Hospital for Maternal and Child Health Care (no. EC-20230726-02), and all participants provided signed written informed consent.

### 2.2 HR-HPV assay and liquid-based cytology test

HPV genotyping was performed using a human papillomavirus (21 types) nucleic acid typing detection kit (Fluorescent PCR) (Hybribio Ltd., Guangzhou, China) according to the manufacturer’s instructions. The process included the following steps: (1) amplification of HPV DNA by polymerase chain reaction (PCR), (2) incubation for hybridization, and (3) enzyme immunoassays for the identification of 21 HPV genotypes. This test included 14 HR-HPV subtypes: HPV 16, 18, 31, 33, 35, 39, 45, 51, 52, 56, 58, 59, 66, and 68.

The main steps of LBC were as follows: (1) expose the cervix, collect the exfoliated cervical epithelial cells using a cervical brush, and rinse them three times in Thin-Prep cell collection bottles (Hologic Inc., MA, USA) with a cytoprotective solution, (2) perform automated thin-section production and detection, (3) use the Bethesda System grading system for cytologic diagnosis as recommended by the International Cancer Society in 2001.

### 2.3 DNA preparation and GYPC methylation assay

The residual specimens in Thin-Prep bottles were sent to the certified Changsha Hoomya Medical Laboratory for *GYPC^m^* testing without clinical information, including symptoms or other testing results. The *GYPC* methylation assay procedure is outlined as follows: (1) Total DNA was extracted from samples, (2) Bisulfite transformation of the DNA was performed, converting unmethylated cytosine into uracil while methylated cytosine remained unchanged; (3) Methylation-specific PCR was conducted on the *GYPC* gene and the internal reference gene Col2A1; and (4) The ΔCp value of the *GYPC^m^* result was calculated as ΔCp = Cp(*GYPC*)-Cp(*Col2A1*); the smaller the ΔCp value, the higher the level of *GYPC^m^*, and vice versa.

### 2.4 Histopathological diagnosis

A colposcopy was performed by a physician in the gynecological colposcopy unit, and multipoint biopsies of suspicious lesions were performed and read independently by two pathologists. Cervical lesions were categorized into normal, CIN1, CIN2, CIN3, and cervical cancer; in case of inconsistency, a third pathologist was asked to read the films.

### 2.5 Statistical analyses

**Figure 2:**
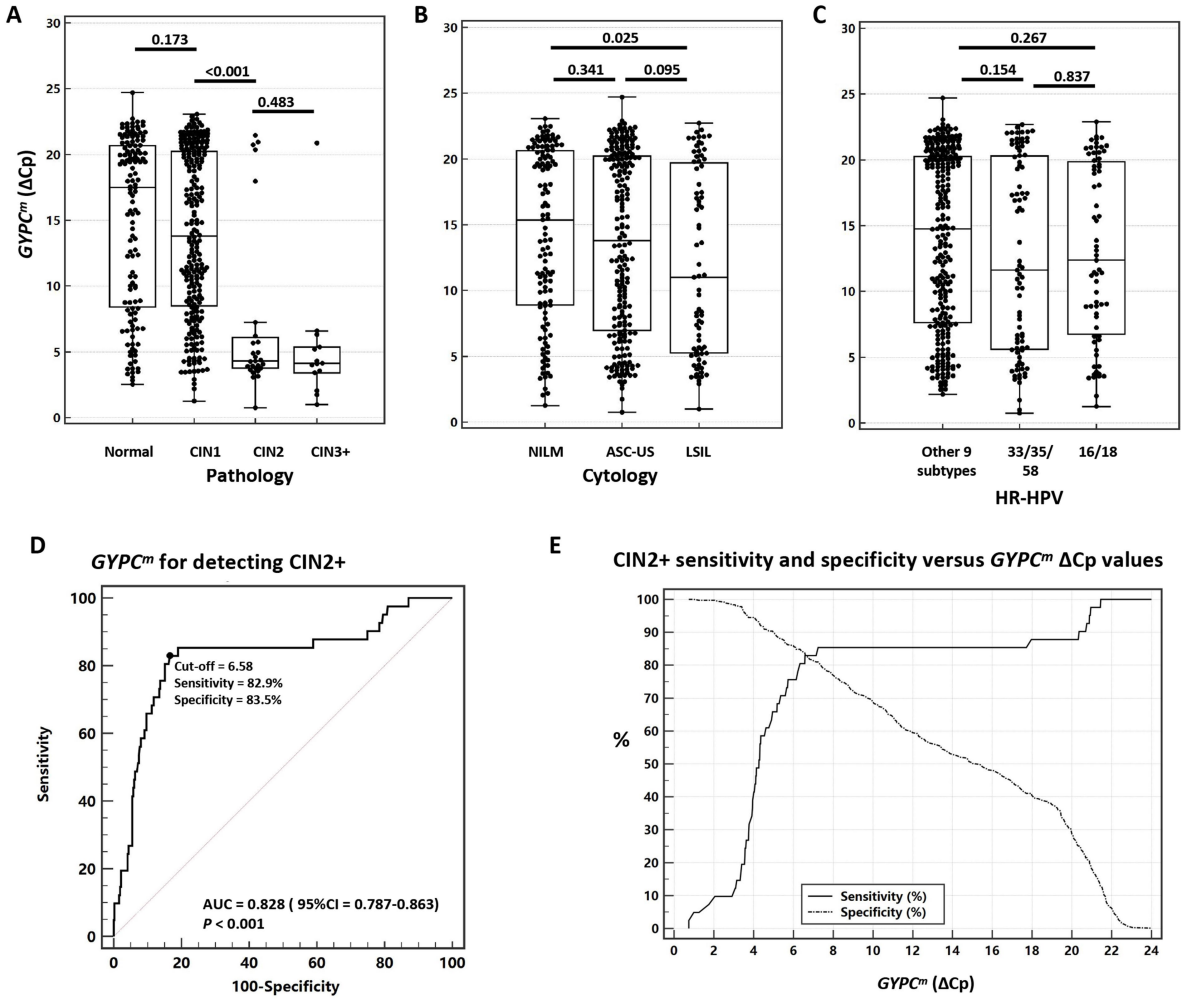
Distribution of *GYPC^m^* (ΔCp) in the study group and ROC plots of *GYPC^m^* for detecting CIN2+. Distribution of *GYPC^m^* (ΔCp) in **(A)** pathology, **(B)** cytology, and **(C)** HR-HPV result groups; **(D)** ROC of *GYPC^m^* for detecting CIN2+. **(E)** CIN2 + sensitivity and specificity versus *GYPC^m^* ΔCp values. CIN1, cervical intraepithelial neoplasia grade 1; CIN2, cervical intraepithelial neoplasia grade 2; CIN2+, cervical intraepithelial neoplasia grade 2 or worse; CIN3, cervical intraepithelial neoplasia grade 3; CIN3+, cervical intraepithelial neoplasia grade 3 or worse; NILM, no intraepithelial lesion or malignancy; ASC-US, atypical squamous cells of undetermined significance; LSIL, low-grade squamous intraepithelial lesion; HR-HPV, high-risk human papillomavirus; AUC, the area under the receiver operating characteristic curve.

**Figure 3:**
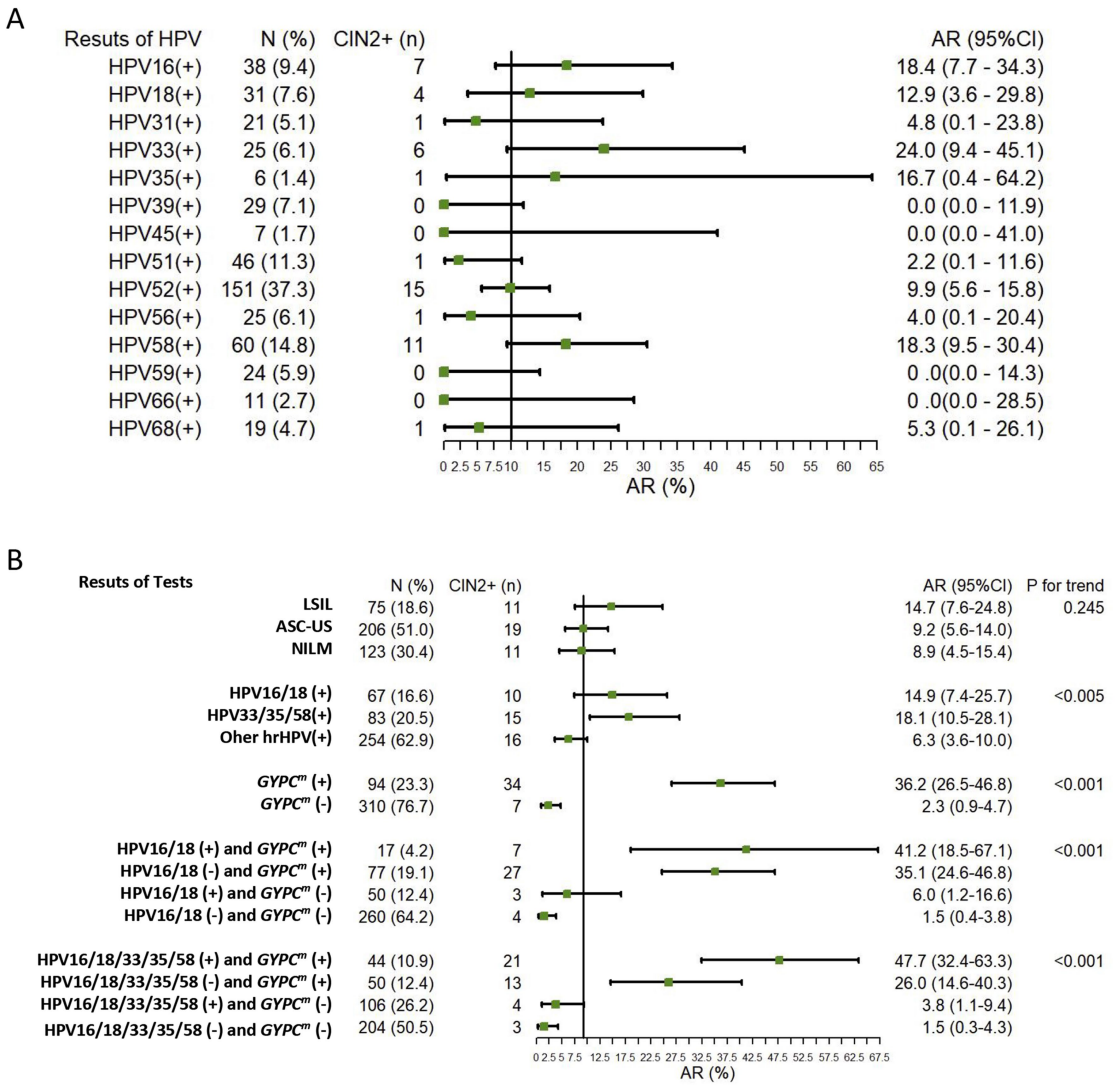
Absolute CIN2 + (*n* = 41) risk of **(A)** HPV test positive and **(B)** other test results. CIN2+, cervical intraepithelial neoplasia grade 2 or worse; *GYPC^m^*, *GYPC* methylation; CI, confidence interval; LBC, liquid-based cytology; ASC-US, atypical squamous cells of undetermined significance; LSIL, low-grade squamous intraepithelial lesion; NILM, no intraepithelial lesion or malignancy; HPV, human papillomavirus; AR, absolute risk.

Continuous variables were recorded as medians (IQR) and categorical variables as frequencies (%). Boxplots were created to observe the distribution of ΔCp for *GYPC^m^*, and the Wilcoxon test was performed to examine the differences between the groups. The ability of *GYPC^m^* to discriminate CIN2+/CIN3 + was estimated using the area under the receiver operating characteristic (ROC) curve (AUC). The critical value of *GYPC^m^* was based on the principle of maximizing Youden’s index for discriminating <CIN2 and CIN2+, with ΔCp ≤ 6.58 interpreted as positive and ΔCp > 6.58 as negative (Figure 2D). Based on the absolute CIN2 + risk of the HPV genotype (Figure 3A), HPV 16, 18, 33, 35, and 58 were grouped. In the test combination, “or” indicates that a positive result is interpreted as positive, while a double negative is interpreted as negative. The absolute CIN2 + risk and its 95% confidence interval (95% CI) were calculated for the different outcomes of each triage strategy. The chi-squared test for trend was used to analyze absolute risk. Sensitivity and specificity for detecting CIN2 + were calculated, and their 95% CIs were estimated using the Clopper–Pearson method. We also calculated the number of colposcopy referrals and their 95% CIs, as well as the number of referrals required to detect one CIN2+. The sensitivity and specificity of the two triage strategies were tested using McNemar’s test. Differences in positive predictive value (PPV) and negative predictive value (NPV) were assessed with the method described by Leisenring et al. (20). All data were analyzed using MedCalc Version 22. Statistical significance was defined as a two-sided *p*-value of < 0.05.

## 3 Results

A total of 404 high-risk HPV-positive women were included in the analysis, with 123 (30.4%) having NILM, 206 (51.0%) having ASC-US, and 75 (18.6%) having LSIL. Of these, 131 had normal cervical findings, 232 had CIN1, 27 had CIN2, 13 had CIN3, and 1 had cervical adenocarcinoma (CA) as the final pathology. The top five HPV genotypes associated with CIN2 + were HPV16, HPV18, HPV33, HPV35, and HPV58 (Figure 3A). The median age of all participants was 43.0 years (IQR = 35.0–52.0), and the median ΔCp of *GYPC^m^* was 13.7 (IQR = 7.0–20.2). Table 1 shows the distribution of clinical findings among cervical lesions.

**Table 1:** Clinical characteristics.

Pathology	Normal *n* (%)	CIN1 *n* (%)	CIN2 *n* (%)	CIN3 + *n* (%)	Total *n* (%)
*N*	131	232	27	14	404
Age Median (IQR)	44.0 (35.0–52.0)	43.5 (36.0–52.5)	39.0 (34.0–50.5)	40.0 (38.0–50.0)	43.0 (35.0–52.0)
HR-HPV group					
HPV16/18(+)	21 (16.1)	36 (15.5)	4 (14.8)	6 (42.9)	67 (16.6)
HPV33/35/58(+)	29 (22.1)	39 (16.8)	12 (44.5)	3 (21.4)	83 (20.5)
Oher hrHPV(+)	81 (61.8)	157 (67.7)	11 (40.7)	5 (35.7)	254 (62.9)
Cytology					
NILM	56 (42.7)	56 (24.1)	7 (25.9)	4 (28.6)	123 (30.4)
ASC-US	66 (50.4)	121 (52.2)	13 (48.1)	6 (42.9)	206 (51.0)
LSIL	9 (6.9)	55 (23.7)	7 (25.9)	4 (28.6)	75 (18.6)
*GYPC^m^* ΔCp median (IQR)	17.5 (8.4–20.7)	13.8 (8.5–20.3)	4.3 (3.7–6.1)	4.1 (3.4–5.4)	13.7 (7.0–20.2)

CIN1, cervical intraepithelial neoplasia grade 1; CIN2, cervical intraepithelial neoplasia grade 2; CIN3, cervical intraepithelial neoplasia grade 3; IQR, interquartile range; HR-HPV, high-risk human papillomavirus; LBC, liquid-based cytology; NILM, no intraepithelial lesion or malignancy; ASC-US, atypical squamous cells of undetermined significance; LSIL, low-grade squamous intraepithelial lesion; *GYPC^m^, GYPC* methylation.

The difference in ΔCp of *GYPC^m^* across HPV genotype groups (HPV16/18, HPV33/35/58, and HPV 31/39/45/51/52/56/59/66/68, other 9 subtypes) was not significant (all *p* > 0.05) (Figure 2C). The difference in ΔCp of *GYPC^m^* between CIN1 and CIN2 was significant (*p* < 0.001) as shown in in Figure 2A. The AUC for *GYPC^m^* to discriminate CIN2 + was 0.828 (95%CI = 0.787–0.863, *p* < 0.001), indicating good discriminatory ability (Figures 2D,E).

The absolute risk of CIN2 + was 36.2% (95%CI = 26.5–46.8%) for *GYPC^m^* (+) and 2.3% (95%CI = 0.9–4.7%) for *GYPC^m^* (−). Four combinations of HPV16/18 and HPV16/18/33/35/58 with *GYPC^m^* testing—(+) (+), (−) (+), (+) (−), and (−) (−)—showed a decreasing trend in absolute CIN2 + risk (*p* < 0.001). HPV genotyping (−) and *GYPC^m^* (−) were associated with minimal absolute CIN2 + risk.

The sensitivities for detecting CIN2 + were 82.9% (95%CI = 67.9–92.8%) for *GYPC^m^*, 24.4% (95%CI = 12.4–40.3%) for HPV16/18, and 61.0% (95%CI = 44.5–75.8%) for HPV16/18/33/35/58, while the specificities were 83.5% (95%CI = 79.2–87.2%), 84.3% (95%CI = 80.1–87.9%), and 65.6% (95%CI = 60.4–70.5%), respectively. *GYPC^m^* demonstrated significantly higher sensitivity compared to HPV16/18 (*p* < 0.001) and higher specificity compared to HPV16/18/33/35/58 (*p* < 0.001) (Table 2).

**Table 2:** Sensitivity, specificity, colposcopy referral percentages, and referrals needed to detect one CIN2+ of triage strategies for HPV-positive women with NILM, ASC-US, and LSIL cytology.

Triage strategy	Sensitivity % (95%CI)	Specificity % (95%CI)	PPV % (95%CI)	NPV % (95%CI)	Colposcopy referral % (95%CI)	Referrals needed to detect one CIN2+
Single triage strategies						
HPV16/18	24.4 (12.4–40.3)	84.3 (80.1–87.9)	14.9 (8.9–24.0)	90.8 (89.2–92.)	16.6 (13.1–20.6)	6.7 (5.2–8.5)
*P*	<0.001	0.839	<0.001	<0.001		
HPV16/18/33/35/58	61.0 (44.5–75.8)	65.6 (60.4–70.5)	16.7 (13.1–21.0)	93.7 (91.0–95.7)	37.1 (32.4–42.0)	6.0 (5.1–7.0)
*P*	0.049	<0.001	<0.001	0.007		
ASC-US+	73.2 (57.0–85.8)	30.9 (26.1–35.9)	10.7 (8.9–12.7)	91.1 (85.7–94.5)	69.6 (64.8–74.1)	9.4 (8.3–10.5)
*P*	0.289	<0.001	<0.001	0.003		
LSIL	26.8 (14.2–42.9)	82.4 (78.0–86.1)	14.7 (9.0–23.0)	90.9 (89.2–92.4)	18.6 (14.9–22.7)	6.8 (5.4–8.5)
*P*	<0.001	0.757	<0.001	<0.001		
^a^ *GYPC^m^*	82.9 (67.9–92.8)	83.5 (79.2–87.2)	36.2 (30.2–42.6)	97.4 (95.6–98.8)	23.3 (19.2–27.3)	2.8 (2.2–3.4)
^b^ *P*	0.727	<0.001	0.002	0.908		
Combined triage strategies						
^c^HPV16/18 or ASC-US+	87.8 (73.8–95.9)	21.8 (17.6–26.4)	11.3 (10.0–12.6)	94.0 (87.2–97.4)	79.2 (74.9–83.1)	8.9 (7.9–9.9)
HPV16/18 or LSIL	51.2 (35.1–67.1)	67.5 (62.4–72.3)	15.1 (11.3–19.9)	92.5 (89.8–94.4)	34.4 (29.8–39.3)	6.6 (5.6–7.8)
*P*	<0.001	<0.001	0.064	0.479		
HPV16/18 or *GYPC^m^*	90.2 (76.9–97.3)	70.5 (65.5–75.2)	25.7 (22.3–29.5)	98.5 (96.2–99.4)	35.6 (32.9–40.5)	3.9 (3.3–4.6)
*P*	>0.999	<0.001	<0.001	0.062		
ASC-US+ or *GYPC^m^*	87.8 (73.8–95.9)	26.2 (21.7–31.0)	11.8 (10.6–13.3)	95.0 (89.1–97.8)	75.2 (70.7–79.3)	8.4 (7.5–9.5)
*P*	>0.999	0.014	0.421	0.713		
^d^ *P*	>0.999	<0.001	<0.001	0.080		
LSIL or *GYPC^m^*	85.4 (70.8–94.4)	70.0 (65.0–74.7)	24.3 (20.8–28.2)	97.7 (95.3–98.9)	35.6 (30.9–40.5)	4.1 (3.5–4.9)
*P*	>0.999	<0.001	<0.001	0.132		
^d^ *P*	0.625	0.916	0.476	0.310		
HPV16/18/33/35/58 or *GYPC^m^*	92.7 (80.1–98.5)	55.4 (50.1–60.6)	19.0 (16.9–21.3)	98.5 (95.7–99.5)	49.5 (44.5–54.5)	5.3 (4.6–6.1)
*P*	0.625	<0.001	<0.001	0.051		
^d^ *P*	>0.999	<0.001	<0.001	0.872		

CIN2+, cervical intraepithelial neoplasia grade 2 or worse; PPV, positive predictive value; NPV, negative predictive value; HPV, human papillomavirus; NILM, no intraepithelial lesion or malignancy; ASC-US, atypical squamous cells of undetermined significance; ASC-US+, atypical squamous cells of undetermined significance or worse; LSIL, low-grade squamous intraepithelial lesion; CI, confidence interval; LBC, liquid-based cytology; *GYPC^m^, GYPC* methylation.

^a^All single triage strategies compared to GYPC^m^; ^b^ P for GYPC^m^ compared with HPV16/18 or ASC-US+; ^c^All combined triage strategies compared to HPV16/18 or ASC-US+; ^d^Compared to HPV16/18 or GYPC^m^.

Sensitivity and specificity were 87.8% (95%CI = 73.8–95.9%) and 21.8% (95%CI = 17.6–26.4%) for HPV16/18 or LBC (ASC-US+); 90.2% (95%CI = 76.9–97.3%) and 70.5% (95%CI = 65.5–75.2%) for HPV16/18 or *GYPC^m^*; and 85.4% (95%CI = 70.8–94.4%) and 70.0% (95%CI = 65.0–74.7%) for LBC (LSIL+) or *GYPC^m^*, respectively. The differences in sensitivity between combined triage strategies were not significant (all *p* > 0.05), except for HPV16/18 or LSIL. The differences in specificity between LSIL or *GYPC^m^* and HPV16/18 or *GYPC^m^* were not significant (*p* = 0.916); however, both were significantly higher than those of the other combined triage strategies (Table 2).

The colposcopy referrals decreased from 79.2% (95%CI = 74.9–83.1%) for HPV16/18 or LBC (ASC-US+) to 49.5% (95%CI = 44.5–54.5%) for HPV16/18/33/35/58 genotyping or *GYPC^m^*, and 35.6% (95%CI = 32.9–40.5%) for HPV16/18 or *GYPC^m^*. The number of colposcopies required to detect one case of CIN2 + decreased from 8.9 for HPV16/18 or LBC (ASC-US+) to 5.3 for HPV16/18/33/35/58 or *GYPC^m^* and 3.9 for HPV16/18 or *GYPC^m^*.

## 4 Discussion

This study explores the risk stratification and triage performance of DNA methylation and viral genotyping in HR-HPV-positive women with either normal or mildly abnormal cytology findings. Current screening strategies include cytology, HR-HPV testing, or both (co-testing), and the various possible combinations of test results lead to complex management, especially for results considered minimally abnormal, defined as results for which it is unclear whether the next step should be a colposcopy or close follow-up. Therefore, further triage management of HPV-positive women with normal or minimal cytological findings is very necessary. *GYPC^m^* was not significantly different between normal and CIN1, CIN2, and CIN3. However, a significant difference was observed between CIN1 and CIN2 (Figure 2A). *GYPC^m^* demonstrated a higher AUC for discriminating between normal/CIN1 and CIN2 + with an AUC of 0.828 (Figure 2D). In addition, the trend of absolute CIN2 + risks for LBC results was not significant (*p* = 0.245). It could be that the small number of CIN2 + cases resulted in a lack of statistical validity or the subjective criteria for cytologic interpretation. However, significant trends were observed for the results of *GYPC^m^*, HPV genotyping, and their combination (Figure 3B). The absolute CIN2 + risk was lowest for cases with double negativity for both HPV genotyping and *GYPC^m^*, at 1.5% (95%CI = 0.4–3.8) and 1.5% (95%CI = 0.3–4.3), respectively.

The number of colposcopy referrals required to detect one case of CIN2 + was lowest at 2.8 for *GYPC^m^* triage, which demonstrated a specificity of 83.5%. The difference in sensitivity between *GYPC^m^* triage and HPV16/18 and LBC (LSIL+) was not significant. Among the combined triage strategies, HPV16/18 and *GYPC^m^* had the lowest number of colposcopy referrals needed to detect one CIN2 + of 3.9 (3.3–4.6). The HPV16/18 or *GYPC^m^* triage strategy was optimal, with the highest specificity of 70.5% (95%CI = 65.5–75.2%). No significant difference was observed between *GYPC^m^* and HPV16/18 or LBC (ASC-US+) in sensitivity (90.2% vs. 87.8%, *p* > 0.999); however, the difference in specificity was significant (70.5% vs. 21.8%, *p* < 0.001).

Numerous studies have reported an association between DNA methylation and cervical lesions (21). In HR-HPV-positive cervical biopsy paraffin tissues, the methylation levels of oncogenes *PAX1* and *ZNF582* increased with the severity of lesions and were positively correlated with the expression of p16 and Ki67 (22). The methylation levels and frequencies of *ANKRD18CP, C13ORF18, EPB41L3, JAM3, SOX1,* and *ZSCAN1* increased with increasing cervical lesion severity (23). In this study, *GYPC^m^* showed a similar trend with increasing severity of lesions (Figure 2A), and the AUC for discriminating between <CIN2 and CIN2 + was 0.828 in HR-HPV-positive women with cytological NILM, ASC-US, and LSIL.

HR-HPV and cytological cervical cancer screening strategies are becoming increasingly popular globally (24–26). HPV16/18-positive and cytologically abnormal, non-16/18 HR-HPV-positive patients are referred for colposcopy (24, 27). In a prospective randomized controlled trial involving two rounds of screening in the Chinese population, colposcopy referrals for the combined HPV and cytology strategy were approximately four times higher than those for cytology screening alone (4). In a previous study, HR-HPV-positive women with ASC-US/LSIL had a CIN3 + risk of 33.1% for *FAM19A4/miR124-2* methylation positivity and 9.8% for negativity (28). This was consistent with the risk observed for the *GYPC^m^*, where the risk of *GYPC^m^* (−) was lower (1.5%).

Gene methylation has gained increasing attention in the diagnosis of cervical cancer and precancerous lesions. Sensitivity and specificity of a six-gene combination (*ASTN1, DLX1, ITGA4, RXFP3, SOX17, and ZNF671*) for detecting CIN2 + in HR-HPV (+) women ranged from 60.8 to 83.0% and 69.9 to 88.4%, respectively (29, 30). The sensitivity of *PAX1* methylation was 83.0–86.2%, and the specificity was 69.9–75.5% in non-16/18 HR-HPV-positive (31, 32). Recent results indicated that the sensitivity and specificity of *GYPC* and *PAX1* methylation for HR-HPV-positive women were similar in our study (19), with *GYPC^m^* having a sensitivity of 82.9% and a specificity of 83.5%. The combination of HPV16/18 with *GYPC^m^* in an “or” combination resulted in a CIN2 + sensitivity of over 90%, a specificity exceeding 70%, and a reduction in colposcopy referrals from 79.2% with the current triage strategy to 35.6%. This reduction would be more pronounced in the absence of colposcopies or in large population screenings.

Limitations of this study include (1) the small sample size, particularly the number of CIN2+, which resulted in wide confidence intervals for CIN2 + sensitivity (Table 2). This limitation may make clinicians hesitant to make decisions owing to insufficient sensitivity, potentially leading to missed diagnoses; (2) no trend was observed in the risk of CIN2 + for NILM, ASC-US, and LSIL, which may be due to insufficiently stringent criteria for cytology interpretation, potentially leading to biased results; (3) the study did not include a follow-up to assess the risk of long-term CIN2 + of the bypassing strategy, making it impossible to determine an evidence-based follow-up period.

Despite the study’s limitations, it was based on clinical practice, providing sufficient support for the potential clinical benefits *GYPC^m^*, or HPV16/18 and LSIL+ combined with *GYPC^m^* triage in HR-HPV-positive women with NILM, ASC-US, and LSIL. This approach could reduce the referral rate of HR-HPV-positive women with mild cytology abnormalities and may improve the detection rate of high-grade lesions in cytology NILM with non-16/18 HR-HPV infection. However, additional information is required to understand the long-term safety of this triage strategy and obtain evidence of follow-up intervals. Thus, future prospective multicenter studies are necessary to provide more evidence-based support for the clinical application of DNA methylation to reduce colposcopy referrals.

## 5 Conclusion

In summary, HPV DNA testing and cytology for cervical cancer screening resulted in high colposcopy referrals for HPV16/18(+) and cytology ASC-US+, leading to the inefficient use of healthcare resources. However, using HPV16/18 or *GYPC^m^* for risk stratification of HR-HPV-positive women with NILM, ASC-US, and LSIL reduced colposcopy referrals from 79.2 to 35.6% while maintaining high CIN2 + sensitivity.

## Data Availability

The raw data supporting the conclusions of this article will be made available by the authors, without undue reservation.
